# Adaptation and Plasticity of *Nannochloropsis* sp. in Response to Seasonal and Geographic Climate Variation

**DOI:** 10.1111/eva.70172

**Published:** 2025-10-24

**Authors:** Isidora Echenique‐Subiabre, Ugbad Farah, Xinyu Lin, Heather Martinez, Ahlem Jebali, Monica R. Mascarenas, Charles J. O'Kelly, Jake Nalley, Shawn R. Starkenburg, Alina A. Corcoran, Jonathan B. Shurin

**Affiliations:** ^1^ University of California San Diego La Jolla California USA; ^2^ New Mexico Consortium Los Alamos New Mexico USA; ^3^ Sandia National Laboratory Albuquerque New Mexico USA; ^4^ Cyanotech Kailua Kona Hawaii USA; ^5^ Qualitas Health Imperial Texas USA; ^6^ Bioscience Division Los Alamos National Laboratory Los Alamos New Mexico USA; ^7^ Department of Biology New Mexico State University Las Cruces New Mexico USA

**Keywords:** common garden experiment, fitness, growth rate, light performance curve, thermal performance curve

## Abstract

Phytoplankton respond to their environment through genetic adaptation and plasticity to maintain fitness. This poses challenges when growing microalgae for industrial applications because, inherently, outdoor mass cultivation may lead to adaptations that alter desirable phenotypic traits and environmental niches. Here, we used common garden experiments to quantify the plasticity and adaptive responses to seasonal and geographic climate variation of *Nannochloropsis*, a microalga commonly used in biotechnology. An initially monoclonal strain was grown outdoors across four locations in Hawaii, Texas, California, and New Mexico. Following 17 and 22 months of cultivation outdoors, we collected samples during winter and summer, respectively, and we compared *strains'* growth from the four sites across temperature and light gradients in the laboratory. Despite hundreds of generations of exposure to divergent climates, with ~20°C and three‐fold differences in daily light intensity, *strains* showed only minor differences in performance. Thermal performance varied more among seasons than sites, whereas light performance varied with both season and site. Our study indicates that *Nannochloropsis* exhibits broad plasticity in response to light and temperature, which may inhibit genetic adaptation in space or time. Highly variable field conditions, with daily and seasonal climate fluctuations, may favor plasticity and prevent the rapid adaptation often seen in laboratory studies of microorganisms in constant environments.

## 1 Introduction

Understanding how microorganisms respond and adapt to environmental variation is a growing priority due to the effects of climate change on ecosystems. Microorganisms' capacity for rapid adaptation to changing environments due to their short generation time, large population size, and high genetic variation makes them unique models to study adaptation and evolution in real time in the laboratory (Lenski 2017). However, few studies have tested the adaptation of microbes in the field under variable conditions in the presence of diverse communities. Standing genetic variation and large population size should promote rapid adaptation and allow for evolutionary rescue to prevent extinction in changing environments (Bell and Collins 2008; Bell 2017; Hufbauer et al. 2015; Gonzalez et al. 2013). It is still unclear how local adaptation and evolutionary rescue contribute to the stability and dynamics of microbial populations and communities over broad spatial and temporal scales.

Organisms may respond to environmental fluctuations through phenotypic plasticity or genetic adaptation (Ghalambor et al. 2007; Merilä and Hendry 2014). Phenotypic plasticity results from changes in gene expression in response to the environment, expanding the range of conditions individuals can tolerate and coexist (Fey et al. 2021), whereas adaptation results from changes in gene frequencies within the population under selection. Plasticity typically occurs during the lifespan of an individual and can be adaptive when positively affecting fitness or maladaptive when moving fitness further away from the optimal phenotype (Ghalambor et al. 2007; Kremer et al. 2018). For example, an adaptive response to higher temperatures will require a greater heat tolerance, whereas a maladaptive response will lower heat tolerance, generating an inappropriate response and decreasing fitness (Campbell‐Staton et al. 2021). Phenotypic plasticity can be measured by exposing individuals from the same population or genotypes to different environments and measuring their phenotype or fitness. Adaptation is revealed by comparing populations or genotypes under the same conditions. Common garden experiments measuring the Genotype by Environment (G×E) interaction reveal the contributions of adaptation and plasticity to organismal response to the environment (Lange et al. 2015; De Villemereuil et al. 2016).

Acclimation to light and temperature is central to the survival and optimization of phytoplankton growth and productivity (Greene et al. 2021; Huesemann et al. 2016; Lee et al. 2015; Quiroz‐Arita et al. 2019; Quiroz et al. 2021; Wang et al. 2019). Growth typically displays a unimodal relationship with light and temperature, with optimal levels of both and declining growth rates with either higher or lower levels. Microalgae undergo photoacclimation by adjusting their light‐harvesting mechanisms based on ambient light intensity, for instance, altering pigment contents or adapting to different light wavelengths, maximizing the use of available spectra (Kumari et al. 2022; Sung et al. 2018). Similarly, thermal acclimation involves dynamic changes at the cellular and molecular levels, such as adjusting lipids and fatty acid composition (Ferrer‐Ledo et al. 2023). Experimental studies in the laboratory have shown rapid adaptation to light and temperature under constant selection (Aranguren‐Gassis et al. 2024). Phytoplankton cope with environmental change through both plasticity and genetic adaptation, and plasticity itself can evolve (Fey et al. 2021). The roles of these two mechanisms under long‐term exposure in fluctuating natural environments are still unknown.

Most research aimed at understanding microalgae evolution, adaptation, and acclimation has been done in laboratory settings with monocultures grown under constant conditions (Buckling et al. 2009; McDonald 2019). These lab‐scale studies reveal the response to constant directional selection but lack the complexity of realistic environments where selective pressures fluctuate in time and indirect effects arise from ecological interactions. In natural environments, environmental changes can occur faster than the time required for acclimation (Torzillo et al. 2003) and selection fluctuates considerably (Bell and Collins 2008), with different factors exerting opposing pressures simultaneously and changing over time (Cohen and Hershberg 2022). Studies of long‐term adaptation and evolutionary processes in the field are greatly needed to understand how microorganisms respond to multiple selection pressures in complex communities (Schaum et al. 2017).

Evolution of microalgae has important implications in cultivation systems for generating bioenergy and other natural products as adaptation to climate may affect growth rate and phenotype. Predicting microbial growth and phenotype under variable field conditions, especially in the face of invasion by weedy taxa, is highly uncertain. Current lab technologies still fail to mimic the field environment and produce unreliable predictions. For example, we recently found large discrepancies between the predictions of a spatio‐temporal model using climate and laboratory‐based strain parameters and our observed field productivity data, even after three different rounds of hindcasting (Echenique‐Subiabre et al. 2023). Changes in growth parameters or phenotype due to plasticity or genotype adaptation are not included in the current models and may be a major cause of poor predictive capacity. Understanding microalgal plasticity and evolution is therefore critical to improve development in biotechnology and productivity optimization.

In our study, we used the single‐celled photosynthetic *Nannochloropsis*, within the Division Eustigmatophyta, a microalga that reproduces asexually and is rich in high‐value compounds including lipids (Converti et al. 2009), proteins (Zhu and Dunford 2013), and polyunsaturated fatty acids (Zhu and Dunford 2013). *Nannochloropsis* shows fast growth (Spolaore et al. 2006) and is used to produce biofuel (Ashour et al. 2019; Edmundson and Huesemann 2015; Liu et al. 2017), food supplements (Kent et al. 2015; Paterson et al. 2023), and aquaculture feeds (Ashour et al. 2019; Li et al. 2020) among other products. *Nannochloropsis* has been successfully grown in outdoor cultivation systems (Rodríguez‐López et al. 2020; Vonshak et al. 2020) and previous research showed that *Nannochloropsis* species exhibit phenotypic plasticity (Zienkiewicz et al. 2020) and rapid adaptation to the environment (Vonshak et al. 2020).

**FIGURE 1:**
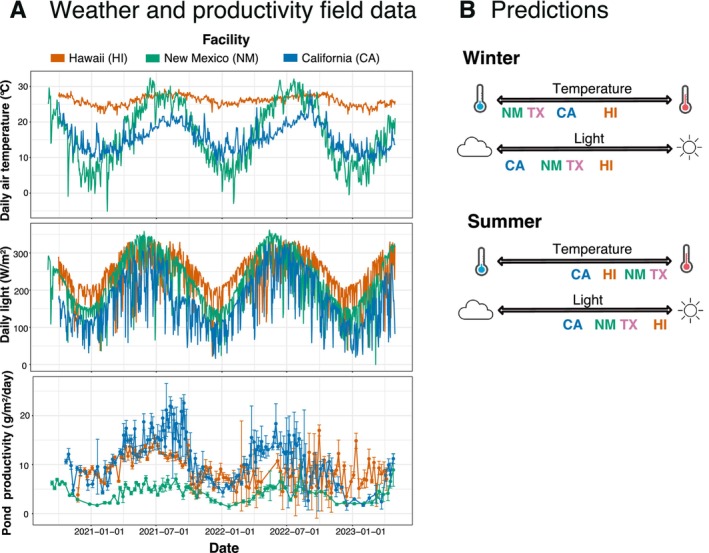
Field data and predictions. (A) Time series of meteorological data and productivity at each of the field sites. Daily averages of the meteorological parameters collected from September 2020 to April 2023 from weather stations within or close to study sites are shown. No data were available for Texas due to intellectual property concerns at the commercial site; however, nearby weather stations indicate that its climate was similar to NM. Productivity is shown as means ± standard deviations. (B) Growth rate predictions for the common garden experiments in a temperature and light gradient, after winter and summer, respectively.

A single strain was seeded in similar raceway ponds maintained under identical protocols in four locations with distinct climate conditions in Hawaii, Texas, California, and New Mexico. We measured the phenotypic plasticity and genetic adaptation among these four strains and their common ancestor. We found large differences in productivity among sites that could not be explained by light and temperature (Echenique‐Subiabre et al. 2023) (Figure 1a). Light and temperature both varied strongly among sites and seasons, with temporal and spatial differences of up to 30°C and three‐fold variation in daily insolation (Figure 1a). This study addresses the following question: how do *Nannochloropsis'* populations isolated from the four sites in winter and summer differ in growth performance across gradients of temperature and light? We predict that strains cultivated in different environments adapt to prevailing conditions of temperature and light, such that strains isolated from warm sites and seasons show temperature‐growth curves shifted to the right relative to colder sites and seasons (Figure 1b), with their optimum growth rates shifted to warmer temperatures. Similarly, we predict that strains isolated from high light sites and seasons will show light‐growth curves shifted to the right when compared to low light sites and seasons (Figure 1b). For instance, we expect that during the winter characterization, New Mexico and Texas field strains will outperform the rest of the strains at low temperatures, and that California field strain will perform better at low light. For the summer trial, we predict that New Mexico and Texas field strains will outperform the rest of the strains at high temperatures, and that the Hawaii field strain will perform better at high light. We expect that the baseline strain's thermal and light niche should not change. Alternatively, seasonally fluctuating selection may prevent local adaptation among sites, or acclimation and plasticity may be sufficient to maintain fitness in a dynamic environment. Our study asks whether genetic evolution of phytoplankton under field conditions where light and temperature change on daily to seasonal time scales affects plasticity and the environmental niche.

## 2 Material and Methods

### 2.1 Microalgae Cultivation and Study Sites

We used the *Nannochloropsis* strain QH25, collected and isolated at Imperial, Texas during 2020 (Sanchez et al. 2022). Cultures were maintained outdoors in open raceway ponds (~200 L) for three consecutive years (2020–2023) at four locations: Kailua‐Kona, Hawaii (19.734593, −156.053035); Imperial, Texas (31.271479, −102.685636); San Diego, California (32.885575, −117.230162); and Las Cruces, New Mexico (32.279262, −106.771913). Three to four replicate ponds were cultivated at each site.

**FIGURE 2:**
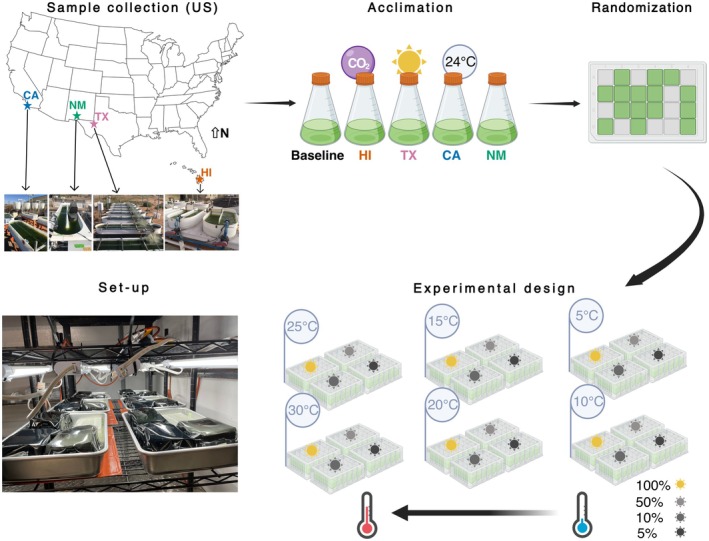
Experimental design and cultivation set‐up. Field cultures and baseline strain were acclimated at control conditions for two growth cycles; subsequently, they were re‐diluted in fresh media, and three replicates per culture were randomly distributed into a 24 deep‐well plate for each treatment. A1 well was filled with sterile water, and the remaining empty wells were filled with culture media. The strains were cultured across all factorial combinations of six temperatures (5°C, 10°C, 15°C, 20°C, 25°C, and 30°C) and four light levels (5%, 10%, 50%, and 100%), resulting in a total of 24 plates (treatment conditions). This image was created using BioRender.

Details on outdoor cultivation and maintenance are described previously (Echenique‐Subiabre et al. 2023). Similarly, the full dataset shown in Figure 1a, which includes the environmental and biological parameters from the cultivation period, is publicly available (Corcoran, Alvarez, et al. 2024). All ponds were running simultaneously in November 2020. Operating pond parameters were similar across sites: Temperature, pH, salinity, and biomass density were monitored Monday through Friday. Pests were checked under a light microscope three times a week. Ponds were harvested and cultures diluted with fresh medium once a target biomass concentration was reached. We measured biomass with two established metrics: optical density at 750 nm (OD_750_) for routine monitoring of pond growth and ash‐free dry weight (AFDW) to calculate pond productivity. A 1 L sample from a single replicate per cultured pond was collected in a Nalgene bottle in 2022 between (i) April 7 and 11th (winter adapted strains; after ~1 year and 5 months of cultivation) and (ii) September 2nd and 13th (summer adapted strains; after ~1 year and 10 months of cultivation). In addition, the initial strain was thawed from cryopreservation each time and used as a baseline to compare the field‐adapted strains. Samples and baseline strain were overnight shipped from each location to San Diego (Figure 2). All strains were re‐inoculated into Erlenmeyer flasks and acclimated to laboratory standard conditions for 14 days (two 7‐day growth cycles) inside an incubator (Thermo Fisher Scientific, Ashville, NC, USA) at 24°C, 1% CO_2_, continuous light (100 μmol m^−2^ s^−1^) and orbital shaking at 90 RPM (VWR Orbital Shaker 3500, Radnor, PA, USA). During the winter experiment version, cultures were acclimated at a final volume of 500 mL. For the summer experiment version, we decreased the acclimation volume to 150 mL due to limited space inside the incubator. Additionally, cultures were treated with bleach (20 ppm on acclimation day 4) to reduce pest loading from the field. This treatment was not needed for the winter experiment nor the baseline strain.

### 2.2 Experimental Design

We tested the performance of each strain compared to the baseline strain in 24‐well deep well plates (VWR, Radnor, PA, USA) exposed to temperature (5°C, 10°C, 15°C, 20°C, 25°C, 30°C) and light (100% = 100 μmol m^−2^ s^−1^, 50% = 50 μmol m^−2^ s^−1^, 10% = 10 μmol m^−2^ s^−1^ and 5% = 5 μmol m^−2^ s^−1^) gradients (Figure 2). The experiment was performed inside a cold room with a target temperature of 5°C. To manipulate temperature, we used heat mats (BRISKHEAT, Columbus, OH, USA). Since the temperature in the cold room was at 5°C, no heat mat was necessary for the lowest temperature. Four deep‐well plates (one for each light treatment) were placed inside six aluminum pans (Nordic Ware Prism, Minneapolis, MN, USA) half‐filled with water sitting on top of the heat mat (Figure 2). We used photographic filters (Renian, China) seated on top of the plates to reduce the light intensity across all wavelengths equally (Brennan et al. 2017).

Each strain was inoculated in three replicate wells 24 times (for each treatment combination of light and temperature) containing a final volume of 7–8 mL of culture at an average starting optical density at 750 nm (OD_750_) of 0.267 and 0.335 for winter and summer experiments, respectively. We randomized the position of the plates and replicates in plates and used sterile distilled water in well A1 of each plate, as well as culture media in empty wells to confirm there was no cross‐contamination across wells. Plates were covered with a Breathe‐Easy sealing membrane (MilliporeSigma, Darmstadt, Germany) to allow for gas exchange. All cultures were grown at ambient CO_2_ (0.04%), in a light: dark (L:D) cycle of 16:8 h regime, and no shaking was provided. We collected samples for optical density on Days 0, 1, 3, and 5. OD_750_ was measured on a 1:1 diluted sample using a Tecan Infinite 200 PRO microplate reader (Tecan, Männedorf, Switzerland). On sampling days, wells were filled with sterile distilled water to replace losses to evaporation.

### 2.3 Calculation of Growth Rates and Statistical Analyses

Cultures were grown for 5 days (Figure S1), and their growth rates were calculated from the slope of OD_750_ increase during the exponential growth phase (i.e., the first 3 days of growth) and used as our measure of performance (or fitness) (Fey et al. 2021). We constructed thermal and light performance curves (TPC and LPC) respectively using generalized additive models (GAMs) from the package “mgcv” (Wood 2023). We quantified the effects of the site (baseline, Hawaii, Texas, California, and New Mexico) and seasonality (winter and summer) on growth rate by comparing models with site‐specific or global TPCs/LPCs. For TPCs, each light treatment was analyzed separately, whereas for LPCs, each temperature treatment was analyzed separately. To select for the best fitting model, we used Restricted Maximum Likelihood (REML) as described previously (Wall et al. 2024). Within each light/temperature treatment and using season or site as the grouping parameters, we applied GAMs comparing three nested models: (i) Model 1: the “simplest” model using a single global smoother fitting all data; (ii) Model 2: a model with a global smoother and a parametric term (i.e., site or season), allowing different intercepts for each group (site or season); and (iii) Model 3: a model providing different smoothers for each group and a parametric term for each group‐specific intercept. Model 1 represents no effect of site or season, or an overall similar TPC/LPC among groups. Model 2 indicates a similar response to temperature/light but with a different offset according to site or season. Model 3 indicates a distinct response to temperature/light with a different group‐specific structure (Pedersen et al. 2019). Models were selected based on the lowest Akaike information criterion (AIC). Their fits, statistical significance of the parametric and smooth terms, and concurvity were assessed using “gam. check,” “anova.gam,” and “concrvty,” respectively, from the packages “mgcv” and “gratia” (Simpson 2024). The anova.gam function performed Wald tests to determine the significance of each parametric and smooth term. We avoided overfitting data and made sure that every model had a Gaussian (bell shape) curve as expected for TPC/LPC when possible, by limiting wiggliness. We used R version 4.2.2 (R Core Team 2022) for statistical analyses. All differences were considered significant when *p* < 0.05.

## 3 Results

### 3.1 Growth Curves

**FIGURE 3:**
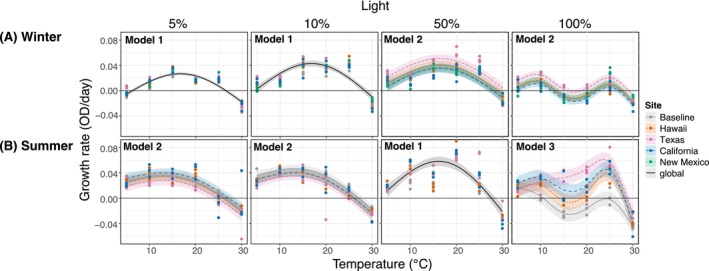
Field strains and baseline growth rates comparison over a temperature gradient at four light treatments after (A) winter and (B) summer season. Points represent the three replicate cultures. Columns separate the four light treatments with temperature treatment on the x‐axis. Lines represent fitted GAM models for significant relationships along with 95% confidence intervals (*p* < 0.05). The continuous black line represents a best fit for Model 1, a global fitted model with no significant differences among strains. Model 1: GAM fits a global smoother to all data. Model 2: GAM has a global smoother allowing for offset intercepts according to site. Model 3: GAM has parametric terms (Site) and separate smoothers for each site. The horizontal black line represents zero growth rate. The best fit model for each plot is shown in the upper left. No summer data is shown for New Mexico culture due to poor growth during the acclimation process.

Growth rates were generally positive from day 0 to 3 at temperatures below 30°C (except for summer field strains at 25°C and 5% light) (Figure S1). We observed a decrease in growth at supraoptimal temperatures, indicating that *Nannochloropsis'* critical thermal maximum is near 30°C (Van Wagenen et al. 2012; Sandnes et al. 2005) (Figure 3). Growth curves were more variable in summer compared to winter, and higher OD_750_ was recorded. This could be related to higher inoculation densities when comparing winter with summer season growth curves. No summer data is shown for New Mexico culture due to poor growth during the acclimation process.

### 3.2 Thermal Performance Curves

We observed significant nonlinear effects of temperature on growth rates (Tables S1 and S2). During winter (Figure 3A), all strains performed similarly, and a unimodal relationship between temperature and growth rate was detected in light treatments from 5% to 50%, with a maximum growth rate between 15°C and 20°C. The same pattern was observed during summer (Figure 3B); however, at 100% light, the shape of the nonlinear relationship was significantly different among strains, with higher growth rates observed for the field strains compared to the baseline. Moreover, the Texas field strain showed higher growth rates than the California and Hawaii field strains between 15°C–25°C. For both seasons, winter and summer, we observed a bi‐modal relationship between growth and temperature at 100% light.

**FIGURE 4:**
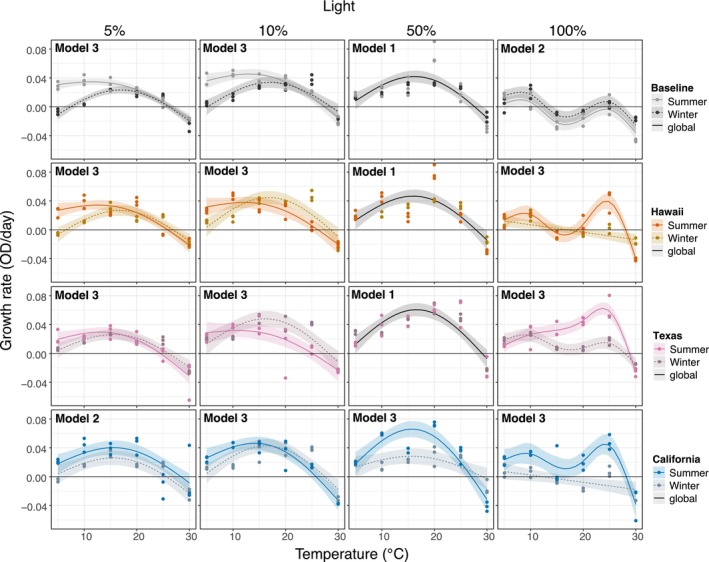
Seasonal growth rates over a temperature gradient at four light treatments for each strain by site. Points represent the three culture replicates. Columns separate the four light treatments. Growth rate is plotted against the temperature gradient. Plotted lines are fitted GAM models for significant relationships along with 95% confidence intervals (*p* < 0.05). The continuous black line represents a global fitted model when no significant differences are detected between seasons. Model 1: GAM fits a global smoother to all data. Model 2: GAM has a global smoother allowing for offset intercepts according to season. Model 3: GAM has parametric terms (Season) and separate smoothers for each season. The horizontal black line represents zero growth rate. The best fit model for each plot is shown in the upper left. No summer data is shown for New Mexico culture due to poor growth during the acclimation process.

We found the largest differences among TPCs between seasons (Figure 4; Tables S3 and S4). Although model 3 was predominantly selected when comparing seasons (Table S3), we found that at low light (5% and 10%), only the Hawaii field‐strain showed a significant distinct pattern between seasons (Table S4), with the summer strain showing faster growth at low temperatures compared to the winter. At 10% light, all field‐strains showed different growth patterns between seasons; however, this was not significant (Table S4). At 50% light, only the California field‐strain showed significant differences among seasons (Table S4), with the summer field‐strain outperforming the winter field‐strain mostly between 10°C and 20°C. At 100% light, all field‐strains' TPCs differed significantly among seasons, with summer field‐strains outperforming the winter field‐strain; the highest growth rates were observed around 25°C. This difference was not observed for the baseline strain.

### 3.3 Light Performance Curves

**FIGURE 5:**
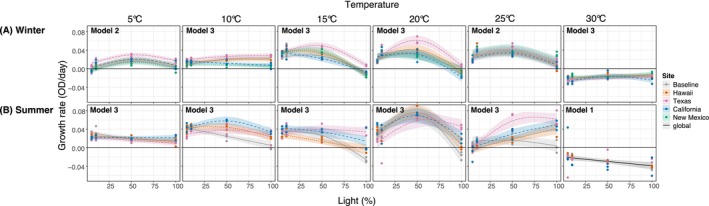
Field strains and baseline growth rates comparison over a light gradient at six temperature treatments after (A) winter and (B) summer season. Points represent the three culture replicates. Columns separate the six temperature treatments. Growth rate is plotted against the light gradient. Continued and dotted lines are fitted GAM models for significant relationships along with 95% confidence intervals (*p* < 0.05). The continuous black line represents a best fit for Model 1, a global fitted model with no significant differences among strains. Model 1: GAM fits a global smoother to all data. Model 2: GAM has a global smoother allowing for offset intercepts according to site. Model 3: GAM has parametric terms (Site) and separate smoothers for each site. The horizontal black line represents zero growth rate. The best fit model for each plot is shown in the upper left. No summer data is shown for New Mexico culture due to poor growth during the acclimation process.

We observed significant nonlinear effects of light on growth rates, with a predominant unimodal relationship (Figure 5 and Tables S5 and S6) and an optimal light level with maximum growth around 50% of the maximum (100 μmol m^−2^ s^−1^). During winter (Figure 5A), light showed different and significant effects on the strains' growth rates depending on the site at 10°C, 15°C, 20°C, and 30°C. However, at 5°C and 25°C, all strains performed similarly, with only different offset intercepts. The Texas field strain showed higher growth rates than the rest of the strains between 5°C–20°C, which became more evident at high light intensities, with peak performance at 50% light. Differences among strains during summer were observed for all temperature treatments from 5°C to 25°C (Figure 5B; Table S6). The baseline strain growth rates declined over the increasing light gradient. California, Hawaii, and Texas field strains overperformed compared to the rest of the strains at 10°C, 20°C, and 25°C, respectively. While model 3 was selected at 20°C, differences among sites were not significant (Table S6). At 30°C, the growth rates of all strains declined similarly with increasing light intensity.

**FIGURE 6:**
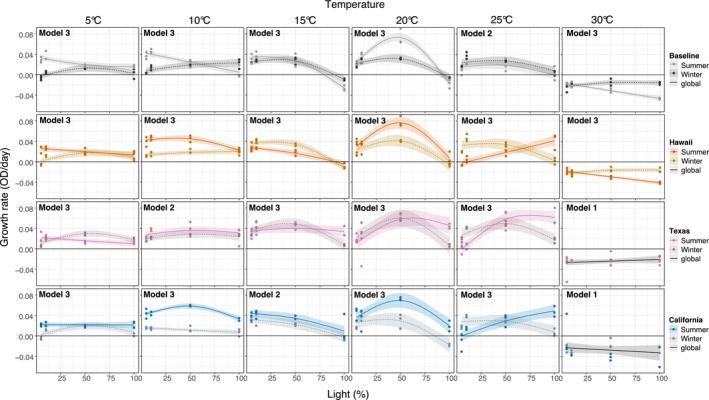
Seasonal growth rates over a light gradient at six temperature treatments for each strain by site. Points represent the three culture replicates. Columns separate the six temperature treatments. Growth rate is plotted against the light gradient. Continued and dotted lines are fitted GAM models for significant relationships along with 95% confidence intervals (*p* < 0.05). The continuous black line represents a global fitted model when no significant differences are detected between seasons. Model 1: GAM fits a global smoother to all data. Model 2: GAM has a global smoother allowing for offset intercepts according to season. Model 3: GAM has parametric terms (Season) and separate smoothers for each season. The horizontal black line represents zero growth rate. The best fit model for each plot is shown in the upper left. No summer data are shown for New Mexico culture due to poor growth during the acclimation process.

LPCs showed significant differences between seasons for each of the strains (Figure 6; Tables S7 and S8). At 5°C and at low light intensities, summer strains outperformed the winter strains, except for Texas field‐strain. This pattern was also observed at 10°C, with summer field‐strains having faster growth over the light gradient compared to the baseline. At 25°C, the summer field‐strains showed an increased tolerance to high light intensity compared to the baseline, however this seasonal difference was only significant for Hawaii field‐strain (Table S8). At 30°C, all strains showed negative growth rates, and no differences among seasons were observed for Texas and California field‐strains.

## 4 Discussion

We examined the adaptation of *Nannochloropsis* to temperature and light regimes in four distinct climate conditions, after 17 months of cultivation outdoors during the winter and subsequently 5 months after, during the summer. Based on rapid adaptive evolution observed in the laboratory (Schlüter et al. 2014; Padfield et al. 2016; Schaum et al. 2016; Yvon‐Durocher et al. 2017), we expected that strains adapted to colder and low light climates over winter would show superior performance under low temperatures and light compared to strains evolved in warmer sites with high light intensities, and vice versa. The growth of *Nannochloropsis* in the field ponds was highly site specific, with each site showing a distinct pattern relating productivity to temperature and light (Echenique‐Subiabre et al. 2023). While we observed seasonal variation, the four populations showed remarkably similar patterns of growth along gradients in temperature (TPCs). The similarity of the thermal niches of the four populations is surprising as the sites varied by around 20°C in the winter and 8°C in the summer, and seasonal differences were up to 30°C in New Mexico (Figure 1). Interestingly, strain performance in relation to light (LPCs) did vary among populations: field strains showed higher tolerance to high light intensities compared to the baseline. Counter intuitively, summer field strains grew faster than winter strains at low temperatures and low light levels; since this was also observed for the baseline strains (at least for 5°C), we do not attribute this to any seasonal effect, instead, to the plasticity of *Nannochloropsis*. Identifying the cause of this contrast requires further study.

Although our experiment encompassed extreme seasonal and geographic climate variation compared to laboratory selection experiments, and over a year of outdoor cultivation, we saw little sign of adaptation in differences among LPCs and TPCs. The present study focuses on TPCs and LPCs as measures of local adaptation. During our field study, we also characterized the phenotypes and genotypes of the field populations every 6 months from the beginning of the outdoor cultivation period until the end (three years of cultivation in total). We used whole genome sequencing (WGS) to detect sequence variation, and flow cytometry to measure cell morphology and chemistry. The phenotype and genotype characterizations found no consistent divergence among field strains (Corcoran, Starkenburg, et al. 2024; Jebali et al. 2025). The lack of evidence for selection for different genotypes among our four sites supports the conclusions of the current study that *Nannochloropsis* did not respond to selection from being grown in dramatically different environments under field conditions.

The lack of detectable and consistent patterns of adaptation to spatial or seasonal climate variation seen in our common garden experiment has several nonmutually exclusive candidate explanations: (1) Broad phenotypic plasticity and acclimation of strains to prevailing conditions reduce the fitness differences among genotypes and thereby prevent genetic adaptation (Scheiner 1993); (2) Not enough generations occurred to observe genetic divergence among field strains compared to the baseline; (3) Genetic diversity within pond populations is maintained by fluctuating conditions so that warm‐ and cold‐ (or high‐ and low‐light) adapted strains coexist within populations, broadening the environmental niches of the populations; (4) Seasonal variation in weather conditions imposed fluctuating selection that changed strength and direction faster than directional selection could produce locally adapted populations; and (5) Interactions with site‐specific members of the microbiome alter the TPC and LPC of *Nannochloropsis*. Below we explore the implications of each of these potential explanations.

### 4.1 Phenotypic Plasticity

The differences in productivity observed at the field sites, in contrast to the small amount of phenotypic or genotypic variation we found, could be attributed to physiological responses originating from *Nannochloropsis'* high phenotypic plasticity (Jebali et al. 2022). Previous studies have shown the high plasticity of *Nannochloropsis* to wide fluctuations of light and temperature stress, for example, by modifying cell size, pigments, and biomass composition in response to temperature and light regimes (Ferrer‐Ledo et al. 2023; Al Jabri et al. 2021). In this context, plasticity can have great implications for adaptive evolution. Plasticity reduces fitness differences among competing genotypes and thereby the effectiveness of selection at changing gene frequencies (Scheiner 1993). Using evolving populations of asexual digital organisms, Lalejini et al. (2021) showed that adaptive plasticity constrains the rate of evolutionary change in fluctuating environments. They found that plastic populations experienced fewer total genetic changes relative to nonplastic populations evolving under the same conditions. These findings are consistent with our results and provide evidence of how plastic organisms can slow genetic changes in fluctuating environments.

### 4.2 Experiment Duration

We estimate that 450–900 generations occurred over the course of our study. We expected to see adaptation to local conditions regarding temperature and light conditions contrasting the different sites; however, we did not. Previous studies have shown mixed results regarding the threshold number of generations needed to observe genetic changes, even in constant environments. This seems to be dependent on the nature of the driver, nutrient conditions, and species specificity. For example, the populations of the green alga *Chlorella vulgaris* showed an evolved tolerance to high temperature (33°C) after ~100 generations of culturing in laboratory conditions (Padfield et al. 2016). The tropical marine diatom, *Chaetoceros simplex*, showed adaptation to above‐optimal temperature (31°C) under nitrogen‐replete conditions after ~200 generations; however, it did not adapt in nitrogen‐limited conditions, suggesting nitrogen limitation could inhibit adaptation to high temperatures (Aranguren‐Gassis et al. 2019). Nevertheless, thermal stress seems to be a strong driver inducing evolution in the lab for microalgae (Aranguren‐Gassis et al. 2024).

On the other hand, over the course of approximately 1000 generations cultured in laboratory conditions, populations of the *Chlamydomonas* green alga showed no observable evolutionary adaptation to an environment with a CO_2_ concentration of 1050 ppm (Collins and Bell 2004). For instance, researchers found no significant improvements in the photophysiological performance or growth of diatoms and coccolithophores after exposure to elevated pCO_2_ for about 65–150 generations. Enhancements in fitness for these species were only noted after extended selection periods spanning several hundred more generations (Chan et al. 2021; Crawfurd et al. 2011; Müller 2017). Contrary to our expectations, it seems that for *Nannochloropsis* to evolve and genotypically change from their original inoculum, thousands of generations will at least be needed; additionally, this will have required a stable (nonfluctuating) and strong environmental pressure. In contrast, one study identified genetic adaptation in our *Nannochloropsis* strain to a more constant and stable cold environment in the laboratory after hundreds of generations (Green et al., unpublished data).

### 4.3 Fluctuating Conditions

Evolution in asexual organisms involves changes in the frequencies of competing clonal lines with different environmental niches. The relative abundances of different clones might change in response to selection, but phenological responses might maintain low fitness strains by preventing competitive exclusion. That is, cold‐adapted strains might slowly decline in their growth throughout the summer but not reach extinction before the arrival of fall and cooler temperatures. A seasonal pattern of succession among different genotypes might broaden the niche of the whole population. That is, different genotypes may have proliferated in our different temperature and light treatments, leading to broader TPCs and LPCs than could be achieved by any single strain. Isolation of monoclonal lines and assessment of their TPCs and LPCs is needed to understand the role of genetic diversity in the environmental niches of phytoplankton adapted to alternate climates.

Interestingly, a bimodal TPC was observed at 100% light across all cultures in both winter and summer, exhibiting two peaks instead of a typical unimodal curve (Figure 3). This result could suggest the existence of dual optima for growth temperatures within our population and/or a pool of diverse genetic coexisting thermotype populations. This bimodal pattern was only apparent at the highest light levels. We hypothesize that there might be a hidden pool of genetic variation in the field strains, allowing subpopulations to coexist in our outdoor ponds. Kling et al. (2023) showed that a population of the cyanobacterium *Synechococcus* was divided into two thermotypes with different optimal growth temperatures, despite their genomes being almost identical (over 99.99% similarity). No genetic variations were found linked to these temperature‐specific phenotypes. However, epigenomic analysis revealed differences in methylation on photosynthesis‐related genes between two strains (Kling et al. 2023). The observed bimodality indicates a sophisticated level of thermal adaptation, potentially mediated by epigenetic regulation rather than genetic differentiation (Kling et al. 2023). The bimodal patterns in *Synechococcus* suggest another possible mechanism behind these odd bimodal TPCs relaying in epigenetic mechanisms; however, the causes in *Nannochloropsis* need further investigation. It is intriguing that the pattern was apparent with every strain and in both seasons only at the highest light level.

### 4.4 Fluctuating Selection

We found larger differences in TPC and LPC between seasons than among field sites despite dramatic geographic differences in climate. Field strains isolated during the summer tended to grow faster than those present in the winter and the baseline when grown at 100% light and 20°C–25°C. Seasonality is an important aspect of environmental variability with a significant role in shaping biodiversity and evolution (Williams et al. 2017). Cyclic phenological changes in light and temperature impose different selective environments than stable conditions (Williams et al. 2017). Adaptive strategies are needed to avoid extinction under seasonality. Many microorganisms have evolved anticipatory physiological adaptations that prepare them before conditions arrive (Nguyen et al. 2021), for example, adjusting their metabolisms to daily cycles of sunlight through circadian rhythms. Over the winter in temperate areas of our study (Texas, California, and New Mexico), ponds experienced cold temperatures and shorter days (i.e., short photoperiods) compared to the warmer summer with longer days. Texas and New Mexico have more seasonal climates, while California is intermediate, and tropical Hawaii is the most stable. We hypothesize that environmental conditions fluctuate faster than beneficial mutations can accumulate to favor adaptive evolution in *Nannochloropsis* field strains (Nguyen et al. 2021). It is likely that selection fluctuated seasonally in our temperate field sites. Interestingly, we saw no evidence of shifts toward higher TPCs or LPCs in tropical Hawaii with a stable environment and very little seasonality in light or temperature (Figure 1). The very small differences in LPCs and TPCs among sites and seasons are surprising given the extreme spatial and climate variation experienced by *Nannochloropsis* throughout our study.

### 4.5 Interactions With Site‐Specific Members of the Phycosphere

Recent advances in the analysis of microalgae's microbiome (the “phycosphere”) show that bacteria can promote algal growth and confer increased tolerance to stressful conditions (Lian et al. 2018). For example, the green algae *Desmodesmus intermedius* experienced protective and enhancing responses to high light and heat in the presence of its native microbiome (Samo et al. 2023). Another study using polar and temperate marine diatoms *Thalassiosira gravida* and *Thalassiosira rotula* , across multiple temperatures and photoperiods, showed positive effects of their microbiomes enhancing maximum growth rates, especially at the margins of their respective niches (Giesler et al. 2023). The phycosphere can modulate the plastic response and can generate and contribute to the host genetic adaptation (Kolodny and Schulenburg 2020). Characterizing pond microbial communities showed dramatic compositional differences among sites and through time in our study (Corcoran, Starkenburg, et al. 2024; Jebali et al. 2025), indicating that the local climate strongly influenced the identities of microbes coexisting with *Nannochloropsis* in our different sites and seasons. Bacteria‐algae interactions may influence the TPCs and LPCs of *Nannochloropsis*, expanding its environmental niche.

The bleach treatment to control contaminating pests in field populations may also have affected the *Nannochloropsis* microbiome and thermal/light adaptation. Bleach is a broad‐spectrum chemical that is known to kill bacteria as well as eukaryotes that would graze differentially on bacteria or *Nannochloropsis*. We observed positive effects of bleach in maintaining *Nannochloropsis* ponds' health during the whole cultivation period and favoring productivity. However, we found that bleach changed the bacterial communities present in our ponds, reducing diversity and favoring certain taxa over others (Jebali et al. 2025). Similar results were obtained in urban landscape ponds, where algal growth was promoted after bleach treatment, but the bacterial community was altered, having differential effects on bacterial taxa (Li et al. 2022). Although bleach was the only treatment used across all sites for pest management, doses were applied as needed locally and seasonally. Consequently, bleach could be considered a factor driving microbial community differentiation across sites and therefore affect thermal adaptation (Jebali et al. 2025).

## 5 Conclusions

Our study provides the analysis of adaptation and plasticity of *Nannochloropsis* in outdoor field conditions subjected to natural environmental fluctuations. Our common garden experiment suggests that differences among strains in TPCs and LPCs are driven mostly by seasonality, and their location of origin has a minor effect on their performance when compared to each other or to the baseline. The lack of apparent adaptation to prevailing field conditions is surprising, as the four field sites experienced dramatically different temperature and light environments. However, the field strains were exposed to highly variable environmental conditions that may have prevented genotype divergence or fixation under directional selection. The lack of adaptation in the field contrasts with studies showing rapid evolution in the lab to alternate conditions. Fluctuating selection, phenotypic plasticity, or ecological interactions with the phycosphere may have prevented adaptation to local climate conditions. Our results suggest that evolution in response to climate may be impeded by evolutionary, genetic, or ecological processes occurring in environments with diverse communities and highly variable conditions. We stress the need for more studies aiming to understand plasticity and adaptation in natural environments, particularly outside the laboratory.

## Disclosure

This report was prepared as an account of work sponsored by an agency of the United States Government. Neither the United States Government nor any agency thereof, nor any of their employees, makes any warranty, express or implied, or assumes any legal liability or responsibility for the accuracy, completeness, or usefulness of any information, apparatus, product, or process disclosed, or represents that its use would not infringe privately owned rights. Reference herein to any specific commercial product, process, or service by trade name, trademark, manufacturer, or otherwise does not necessarily constitute or imply its endorsement, recommendation, or favoring by the United States Government or any agency thereof. The views and opinions of authors expressed herein do not necessarily state or reflect those of the United States Government or any agency thereof.

## Conflicts of Interest

The authors declare no conflicts of interest.

## Supporting information


**Figure S1:** Winter and summer season growth curves over a temperature gradient at four light treatments. Points represent the three culture replicates average; error bars are standard deviation. Winter samples are represented on top of summer for comparison. Columns show the six temperature treatments and rows show the four light treatments. No summer data are shown for New Mexico culture due to culture health issues during the acclimation process. Day 1 for the temperature‐light treatment combinations: 30°C‐100%, 30°C‐50%, 20°C‐10% and 20°C‐5% is missing due to a measuring error, the reason why OD_750_ values were discarded.
**Table S1:** Model selection for thermal performance curve comparison among sites with candidate GAM models assessed at each light treatment, corresponding to 5%, 10%, 50%, and 100%.
**Table S2:** Generalized additive models (GAM) testing site and factor‐smooth interaction effects on growth rate measured at winter and summer for thermal performance curve. Separate smoothers were fit for each site, and ANOVA tables were generated by anova.gam().
**Table S3:** Model selection for thermal performance curve comparison between seasons with candidate GAM models assessed at each light treatment, corresponding to 5%, 10%, 50%, and 100%.
**Table S4:** Generalized additive models (GAM) testing season and factor‐smooth interaction effects on growth rate measured at each site for thermal performance curve. Separate smoothers were fit for each season, and ANOVA tables were generated by anova.gam().
**Table S5:** Model selection for light performance curve comparison among sites with candidate GAM models assessed at each temperature treatment, corresponding to 5°C, 10°C, 15°C, 20°C, 25°C, and 30°C.
**Table S6:** Generalized additive models (GAM) testing site and factor‐smooth interaction effects on growth rate measured at winter and summer for light performance curve. Separate smoothers were fit for each site, and ANOVA tables were generated by anova.gam().
**Table S7:** Model selection for light performance curve comparison between seasons with candidate GAM models assessed at each temperature treatment, corresponding to 5°C, 10°C, 15°C, 20°C, 25°C, and 30°C.
**Table S8:** Generalized additive models (GAM) testing season and factor‐smooth interaction effects on growth rate measured at each site for light performance curve. Separate smoothers were fit for each season, and ANOVA tables were generated by anova.gam().

## Data Availability

The data files and R code that support the findings of this study are openly available in the DRYAD repository (https://doi.org/10.5061/dryad.rv15dv4mz).
