# Supplementary material for: Adaptation and Plasticity of Nannochloropsis sp. in Response to Seasonal and Geographic Climate Variation
Source: Evol Appl. 2025 Oct 24;18(10):e70172. doi: 10.1111/eva.70172 (PMC12552136; doi:10.1111/eva.70172)
Supplement: Supplementary file 1 — Figure S1: Winter and summer season growth curves over a temperature gradient at four light treatments. Points represent the three culture replicates average; error bars are standard deviation. Winter samples are represented on top of summer for comparison. Columns show the six temperature treatments and rows show the four light treatments. No summer data are shown for New Mexico culture due to culture health issues during the acclimation process. Day 1 for the temperature‐light treatment combinations: 30°C‐100%, 30°C‐50%, 20°C‐10% and 20°C‐5% is missing due to a measuring error, the reason why OD750 values were discarded. Table S1: Model selection for thermal performance curve comparison among sites with candidate GAM models assessed at each light treatment, corresponding to 5%, 10%, 50%, and 100%. Table S2: Generalized additive models (GAM) testing site and factor‐smooth interaction effects on growth rate measured at winter and summer for thermal performance curve. Separate smoothers were fit for each site, and ANOVA tables were generated by anova.gam(). Table S3: Model selection for thermal performance curve comparison between seasons with candidate GAM models assessed at each light treatment, corresponding to 5%, 10%, 50%, and 100%. Table S4: Generalized additive models (GAM) testing season and factor‐smooth interaction effects on growth rate measured at each site for thermal performance curve. Separate smoothers were fit for each season, and ANOVA tables were generated by anova.gam(). Table S5: Model selection for light performance curve comparison among sites with candidate GAM models assessed at each temperature treatment, corresponding to 5°C, 10°C, 15°C, 20°C, 25°C, and 30°C. Table S6: Generalized additive models (GAM) testing site and factor‐smooth interaction effects on growth rate measured at winter and summer for light performance curve. Separate smoothers were fit for each site, and ANOVA tables were generated by anova.gam(). Table S7: Model selection [file EVA-18-e70172-s001.docx]

**Supplementary material**


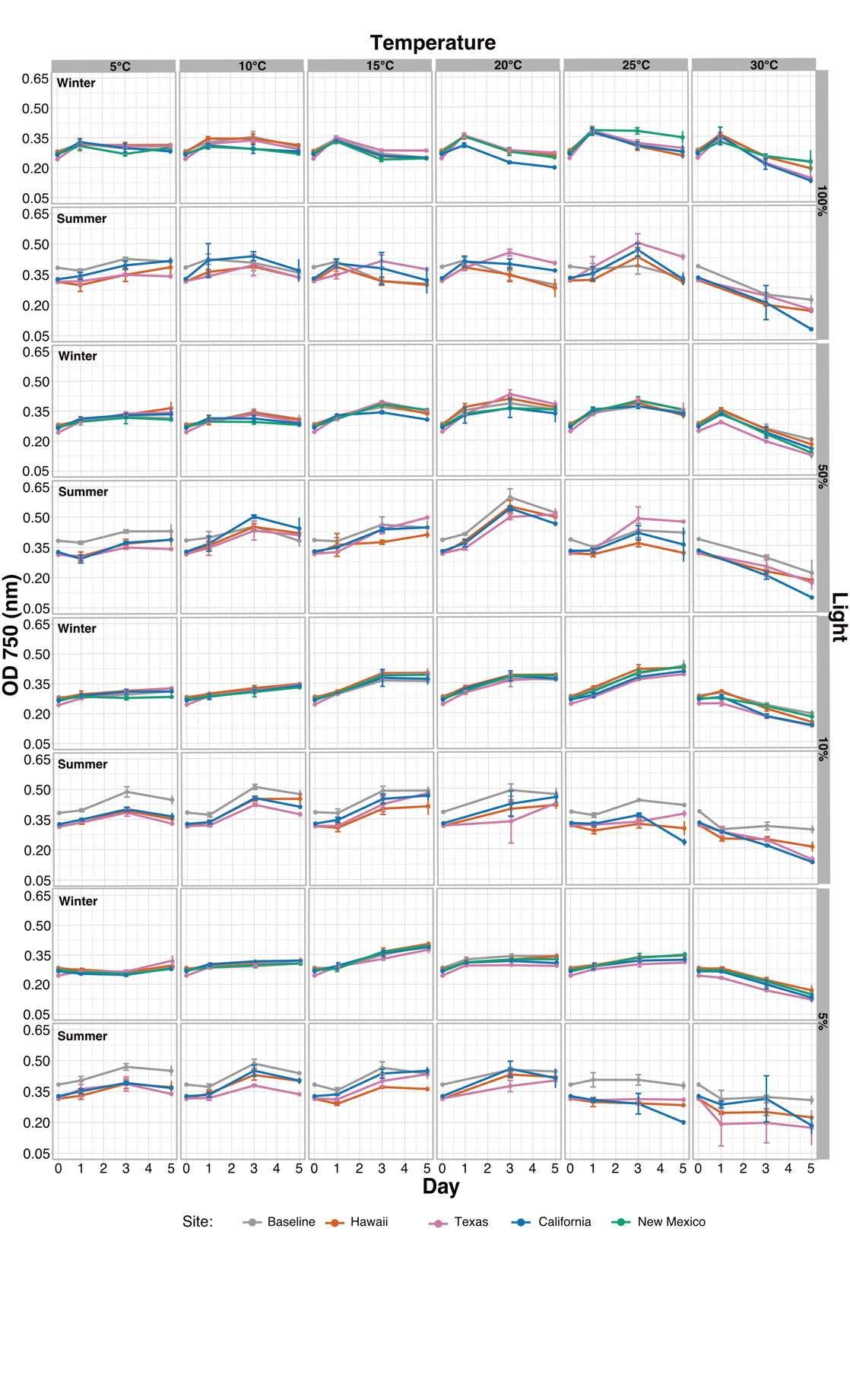


**Figure S1: Winter and summer season growth curves over a temperature gradient at four light treatments.** Points represent the three culture replicates average; error bars are standard deviation. Winter samples are represented on top of summer for comparison. Columns show the six temperature treatments and rows show the four light treatments. No summer data is shown for New Mexico culture due to culture health issues during the acclimation process. Day 1 for the temperature-light treatment combinations: 30°C-100%, 30°C-50%, 20°C-10% and 20°C-5% is missing due to a measuring error, the reason why OD_750_ values were discarded.

**Table S1.** Model selection for thermal performance curve comparison among sites with candidate GAM models assessed at each light treatment, corresponding to 5, 10, 50, and 100%.

| Season | Light | Model | df | AIC |
| --- | --- | --- | --- | --- |
| Winter | 5% | ~Site + s(Temperature, by= Site) | 16 | -601.9 |
|  |  | ~Site + s(Temperature) | 8 | -609.1 |
|  |  | **~s(Temperature)** | **4** | **-612.6** |
|  | 10% | ~Site + s(Temperature, by= Site) | 16 | -496.2 |
|  |  | ~Site + s(Temperature) | 8 | -504.9 |
|  |  | **~s(Temperature)** | **4** | **-508.5** |
|  | 50% | ~Site + s(Temperature, by= Site) | 16 | -510.1 |
|  |  | **~Site + s(Temperature)** | **9** | **-517** |
|  |  | ~s(Temperature) | 4 | -507.6 |
|  | 100% | ~Site + s(Temperature, by= Site) | 18.6 | -561.4 |
|  |  | **~Site + s(Temperature)** | **10.9** | **-599.8** |
|  |  | ~s(Temperature) | 6.9 | -576.4 |
| Summer | 5% | ~Site + s(Temperature, by= Site) | 13 | -395.5 |
|  |  | **~Site + s(Temperature)** | **7** | **-406.1** |
|  |  | ~s(Temperature) | 4 | -404.3 |
|  | 10% | ~Site + s(Temperature, by= Site) | 13 | -429.2 |
|  |  | **~Site + s(Temperature)** | **7** | **-433.9** |
|  |  | ~s(Temperature) | 4 | -429.7 |
|  | 50% | ~Site + s(Temperature, by= Site) | 13 | -343.1 |
|  |  | ~Site + s(Temperature) | 7 | -350.2 |
|  |  | **~s(Temperature)** | **4** | **-352.9** |
|  | 100% | **~Site + s(Temperature, by= Site)** | **24.3** | **-410.5** |
|  |  | ~Site + s(Temperature) | 9.9 | -389.9 |
|  |  | ~s(Temperature) | 6.9 | -346.7 |

**Model 3: Site + s(Temperature, by= Site) GAM has parametric terms (Site) and separate smoothers for each site. Model 2: Site + s(Temperature) GAM has a global smoother allowing for offset intercepts according to site. Model 1: s(Temperature) GAM fits a global smoother to all data. Bold represents the selected models.*

**Table S2.** Generalized additive models (GAM) testing site and factor-smooth interaction effects on growth rate measured at winter and summer for thermal performance curve. Separate smoothers were fit for each site, and ANOVA tables were generated by anova.gam().

| Season | Light | Effect | df/edf | Ref.df | F | p-value |
| --- | --- | --- | --- | --- | --- | --- |
| Winter | 5% | s(Temperature) | 1.997 | 2.000 | 177.9 | **< 2e-16** |
|  | 10% | s(Temperature) | 1.994 | 2.000 | 81.76 | **< 2e-16** |
|  | 50% | Site | 4 | - | 4.41 | **0.00278** |
|  |  | s(Temperature) | 1.991 | 2.000 | 66.85 | **< 2e-16** |
|  | 100% | Site | 4 | - | 8.336 | **1.13e-05** |
|  |  | s(Temperature) | 4.882 | 4.993 | 37.5 | **< 2e-16** |
| Summer | 5% | Site | 3 | - | 2.488 | 0.068 |
|  |  | s(Temperature) | 1.979 | 2.000 | 70.73 | **< 2e-16** |
|  | 10% | Site | 3 | - | 3.33 | **0.0247** |
|  |  | s(Temperature) | 1.99 | 2.000 | 141.4 | **< 2e-16** |
|  | 50% | s(Temperature) | 1.991 | 2.000 | 63.94 | **< 2e-16** |
|  | 100% | Site | 3 | - | 32.56 | **1.07e-11** |
|  |  | s(Temperature): Baseline | 4.335 | 4.801 | 12.02 | **7.11e-07** |
|  |  | s(Temperature): Hawaii | 4.708 | 4.959 | 16.94 | **< 2e-16** |
|  |  | s(Temperature): Texas | 4.541 | 4.901 | 20.36 | **< 2e-16** |
|  |  | s(Temperature): California | 4.679 | 4.951 | 15.39 | **< 2e-16** |

**Site indicates the parametric term in GAM, s(Temperature) is the smooth term for each site. df/edf column indicates either df (degrees of freedom) for parametric terms or edf (effective degrees of freedom) for smoother terms; Ref.df = reference degree of freedom, where dashes indicate NA for parametric terms. Significant effects (p<0.05) are in bold. No summer data is shown for New Mexico culture due to health issues during the acclimation process.*

**Table S3.** Model selection for thermal performance curve comparison between seasons with candidate GAM models assessed at each light treatment, corresponding to 5, 10, 50, and 100%.

| Site | Light | Model | df | AIC |
| --- | --- | --- | --- | --- |
| Baseline | 5% | **~Season + s(Temperature, by= Season)** | **7** | **-250.5** |
|  |  | ~Season + s(Temperature) | 5 | -223 |
|  |  | ~s(Temperature) | 4 | -209.5 |
|  | 10% | **~Season + s(Temperature, by= Season)** | **7** | **-219** |
|  |  | ~Season + s(Temperature) | 5 | -204.6 |
|  |  | ~s(Temperature) | 4 | -200.3 |
|  | 50% | ~Season + s(Temperature, by= Season) | 7 | -190.3 |
|  |  | ~Season + s(Temperature) | 5 | -189.2 |
|  |  | **~s(Temperature)** | **4** | **-190.6** |
|  | 100% | ~Season + s(Temperature, by= Season) | 8.8 | -215.8 |
|  |  | **~Season + s(Temperature)** | **7.9** | **-218.7** |
|  |  | ~s(Temperature) | 6.9 | -210.6 |
| Hawaii | 5% | **~Season + s(Temperature, by= Season)** | **7** | **-222.4** |
|  |  | ~Season + s(Temperature) | 5 | -209.7 |
|  |  | ~s(Temperature) | 4 | -207 |
|  | 10% | **~Season + s(Temperature, by= Season)** | **7** | **-206.3** |
|  |  | ~Season + s(Temperature) | 5 | -196.5 |
|  |  | ~s(Temperature) | 4 | -198.3 |
|  | 50% | ~Season + s(Temperature, by= Season) | 7 | -180.4 |
|  |  | ~Season + s(Temperature) | 5 | -180.9 |
|  |  | **~s(Temperature)** | **4** | **-182.6** |
|  | 100% | **~Season + s(Temperature, by= Season)** | **8.9** | **-220.3** |
|  |  | ~Season + s(Temperature) | 7.9 | -205.2 |
|  |  | ~s(Temperature) | 6.9 | -203.9 |
| Texas | 5% | **~Season + s(Temperature, by= Season)** | **7** | **-219.3** |
|  |  | ~Season + s(Temperature) | 5 | -215.6 |
|  |  | ~s(Temperature) | 4 | -217.6 |
|  | 10% | **~Season + s(Temperature, by= Season)** | **7** | **-191.8** |
|  |  | ~Season + s(Temperature) | 5 | -189 |
|  |  | ~s(Temperature) | 4 | -187.2 |
|  | 50% | ~Season + s(Temperature, by= Season) | 7 | -178.7 |
|  |  | ~Season + s(Temperature) | 5 | -181.8 |
|  |  | **~s(Temperature)** | **4** | **-183.8** |
|  | 100% | **~Season + s(Temperature, by= Season)** | **12.7** | **-228.6** |
|  |  | ~Season + s(Temperature) | 7.8 | -195.2 |
|  |  | ~s(Temperature) | 6.7 | -186.3 |
| California | 5% | ~Season + s(Temperature, by= Season) | 7 | -186.4 |
|  |  | **~Season + s(Temperature)** | **5** | **-187.1** |
|  |  | ~s(Temperature) | 4 | -182.4 |
|  | 10% | **~Season + s(Temperature, by= Season)** | **7** | **-201.6** |
|  |  | ~Season + s(Temperature) | 5 | -196.8 |
|  |  | ~s(Temperature) | 4 | -198.7 |
|  | 50% | **~Season + s(Temperature, by= Season)** | **7** | **-190** |
|  |  | ~Season + s(Temperature) | 5 | -178 |
|  |  | ~s(Temperature) | 4 | -175.5 |
|  | 100% | **~Season + s(Temperature, by= Season)** | **8.9** | **-192.6** |
|  |  | ~Season + s(Temperature) | 7.9 | -188.7 |
|  |  | ~s(Temperature) | 6.8 | -171.3 |

**Model 3: Season + s(Temperature, by= Season) GAM has parametric terms (Season) and separate smoothers for each season. Model 2: Season + s(Temperature) GAM has a global smoother allowing for offset intercepts according to season. Model 1: s(Temperature) GAM fits a global smoother to all data. Bold represents the selected models. No data is shown for New Mexico culture due to health issues during the summer acclimation process.*

**Table S4.** Generalized additive models (GAM) testing season and factor-smooth interaction effects on growth rate measured at each site for thermal performance curve. Separate smoothers were fit for each season, and ANOVA tables were generated by anova.gam().

| Site | Light | Effect | df/edf | Ref.df | F | p-value |
| --- | --- | --- | --- | --- | --- | --- |
| Baseline | 5% | Season | 1 | - | 38.84 | **7.25e-07** |
|  |  | s(Temperature): winter | 1.989 | 2.000 | 46.86 | **< 2e-16** |
|  |  | s(Temperature): summer | 1.968 | 1.999 | 65.10 | **< 2e-16** |
|  | 10% | Season | 1 | - | 9.459 | **0.00445** |
|  |  | s(Temperature): winter | 1.974 | 1.999 | 18.64 | **5.66e-06** |
|  |  | s(Temperature): summer | 1.961 | 1.998 | 36.31 | **< 2e-16** |
|  | 50% | s(Temperature) | 1.978 | 2.000 | 26.18 | **< 2e-16** |
|  | 100% | Season | 1 | - | 9.268 | **0.00488** |
|  |  | s(Temperature) | 4.633 | 4.936 | 16.32 | **< 2e-16** |
| Hawaii | 5% | Season | 1 | - | 6.568 | **0.0156** |
|  |  | s(Temperature): winter | 1.976 | 1.999 | 21.14 | **2.12e-06** |
|  |  | s(Temperature): summer | 1.955 | 1.998 | 34.13 | **< 2e-16** |
|  | 10% | Season | 1 | - | 0.227 | 0.637 |
|  |  | s(Temperature): winter | 1.976 | 1.999 | 21.64 | **1.78e-06** |
|  |  | s(Temperature): summer | 1.933 | 1.995 | 24.34 | **6.13e-07** |
|  | 50% | s(Temperature) | 1.974 | 1.999 | 23.6 | **4.91e-07** |
|  | 100% | Season | 1 | - | 4.262 | **0.0483** |
|  |  | s(Temperature): winter | 1.000 | 1.000 | 14.08 | **0.000813** |
|  |  | s(Temperature): summer | 4.776 | 4.975 | 22.54 | **< 2e-16** |
| Texas | 5% | Season | 1 | - | 0.051 | 0.823 |
|  |  | s(Temperature): winter | 1.966 | 1.999 | 19.36 | **3.78e-06** |
|  |  | s(Temperature): summer | 1.963 | 1.999 | 37.13 | **< 2e-16** |
|  | 10% | Season | 1 | - | 4.052 | 0.0531 |
|  |  | s(Temperature): winter | 1.964 | 1.999 | 16.07 | **1.63e-05** |
|  |  | s(Temperature): summer | 1.875 | 1.984 | 14.65 | **2.67e-05** |
|  | 50% | s(Temperature) | 1.984 | 2.000 | 32.56 | **< 2e-16** |
|  | 100% | Season | 1 | - | 28.29 | **1.64e-05** |
|  |  | s(Temperature): winter | 4.284 | 4.772 | 9.163 | **6.13e-05** |
|  |  | s(Temperature): summer | 4.737 | 4.966 | 37.128 | **< 2e-16** |
| California | 5% | Season | 1 | - | 6.562 | **0.0153** |
|  |  | s(Temperature) | 1.961 | 1.998 | 17.38 | **6.65e-06** |
|  | 10% | Season | 1 | - | 0.126 | 0.725 |
|  |  | s(Temperature): winter | 1.974 | 1.999 | 21.56 | **1.77e-06** |
|  |  | s(Temperature): summer | 1.971 | 1.999 | 36.94 | **< 2e-16** |
|  | 50% | Season | 1 | - | 6.133 | **0.0191** |
|  |  | s(Temperature): winter | 1.870 | 1.983 | 4.121 | **0.0209** |
|  |  | s(Temperature): summer | 1.983 | 2.000 | 39.493 | **< 2e-16** |
|  | 100% | Season | 1 | - | 23.43 | **4.49e-05** |
|  |  | s(Temperature): winter | 1.000 | 1.000 | 7.128 | **0.0127** |
|  |  | s(Temperature): summer | 4.565 | 4.912 | 11.055 | **1.19e-05** |

**Season indicates the parametric term in GAM, s(Temperature) is the smooth term for each season. df/edf column indicates either df (degrees of freedom) for parametric terms or edf (effective degrees of freedom) for smoother terms; Ref.df = reference degree of freedom, where dashes indicate NA for parametric terms. Significant effects (p<0.05) are in bold. No data is shown for New Mexico culture due to health issues during the summer acclimation process.*

**Table S5.** Model selection for light performance curve comparison among sites with candidate GAM models assessed at each temperature treatment, corresponding to 5, 10, 15, 20, 25, and 30°C.

| Season | Temperature | Model | df | AIC |
| --- | --- | --- | --- | --- |
| winter | 5°C | ~Site + s(Light, by= Site) | 16 | -412.9 |
|  |  | **~Site + s(Light)** | **8** | **-426.4** |
|  |  | ~s(Light) | 4 | -395.5 |
|  | 10°C | **~Site + s(Light, by= Site)** | **13.5** | **-454.8** |
|  |  | ~Site + s(Light) | 7.9 | -433.8 |
|  |  | ~s(Light) | 3.8 | -406.8 |
|  | 15°C | **~Site + s(Light, by= Site)** | **15.9** | **-421.6** |
|  |  | ~Site + s(Light) | 8 | -409.6 |
|  |  | ~s(Light) | 4 | -391.4 |
|  | 20°C | **~Site + s(Light, by= Site)** | **16** | **-380.8** |
|  |  | ~Site + s(Light) | 8 | -372.6 |
|  |  | ~s(Light) | 4 | -366.9 |
|  | 25°C | ~Site + s(Light, by= Site) | 15.7 | -367 |
|  |  | **~Site + s(Light)** | **8** | **-367.4** |
|  |  | ~s(Light) | 4 | -361.7 |
|  | 30°C | **~Site + s(Light, by= Site)** | **14.1** | **-445.8** |
|  |  | ~Site + s(Light) | 7.9 | -445.3 |
|  |  | ~s(Light) | 3.8 | -438.3 |
| summer | 5°C | **~Site + s(Light, by= Site)** | **10.1** | **-348.2** |
|  |  | ~Site + s(Light) | 6.9 | -344.5 |
|  |  | ~s(Light) | 3.9 | -341.5 |
|  | 10°C | **~Site + s(Light, by= Site)** | **11.9** | **-308.8** |
|  |  | ~Site + s(Light) | 7 | -294.1 |
|  |  | ~s(Light) | 4 | -281.7 |
|  | 15°C | **~Site + s(Light, by= Site)** | **11.5** | **-292.3** |
|  |  | ~Site + s(Light) | 7 | -270.9 |
|  |  | ~s(Light) | 4 | -257.6 |
|  | 20°C | **~Site + s(Light, by= Site)** | **13** | **-257.1** |
|  |  | ~Site + s(Light) | 7 | -236.9 |
|  |  | ~s(Light) | 4 | -239.3 |
|  | 25°C | **~Site + s(Light, by= Site)** | **11.5** | **-276.2** |
|  |  | ~Site + s(Light) | 7 | -246 |
|  |  | ~s(Light) | 4 | -242.5 |
|  | 30°C | ~Site + s(Light, by= Site) | 9.8 | -254.8 |
|  |  | ~Site + s(Light) | 6 | -253.1 |
|  |  | **~s(Light)** | **3** | **-258.6** |

**Model 3: Site + s(Temperature, by= Site) GAM has parametric terms (Site) and separate smoothers for each site. Model 2: Site + s(Light) GAM has a global smoother allowing for offset intercepts according to site. Model 1: s(Light) GAM fits a global smoother to all data. Bold represents the selected models.*

**Table S6.** Generalized additive models (GAM) testing site and factor-smooth interaction effects on growth rate measured at winter and summer for light performance curve. Separate smoothers were fit for each site, and ANOVA tables were generated by anova.gam().

| Season | Temperature | Effect | df/edf | Ref.df | F | p-value |
| --- | --- | --- | --- | --- | --- | --- |
| winter | 5°C | Site | 4 | - | 12.09 | **4.7e-07** |
|  |  | s(Light) | 1.984 | 2.000 | 34.88 | **< 2e-16** |
|  | 10°C | Site | 4 | - | 16.26 | **1.72e-08** |
|  |  | s(Light): Baseline | 1.804 | 1.962 | 12.782 | **0.000132** |
|  |  | s(Light): Hawaii | 1.467 | 1.716 | 3.088 | 0.118882 |
|  |  | s(Light): Texas | 1.568 | 1.814 | 5.379 | **0.025271** |
|  |  | s(Light): California | 1.000 | 1.000 | 5.845 | **0.019463** |
|  |  | s(Light): New Mexico | 1.000 | 1.000 | 1.592 | 0.213192 |
|  | 15°C | Site | 4 | - | 9.881 | **7.76e-06** |
|  |  | s(Light): Baseline | 1.95 | 1.997 | 34.99 | **< 2e-16** |
|  |  | s(Light): Hawaii | 1.959 | 1.998 | 62 | **< 2e-16** |
|  |  | s(Light): Texas | 1.975 | 1.999 | 36.67 | **< 2e-16** |
|  |  | s(Light): California | 1.765 | 1.945 | 40.37 | **< 2e-16** |
|  |  | s(Light): New Mexico | 1.973 | 1.999 | 74.92 | **< 2e-16** |
|  | 20°C | Site | 4 | - | 4.351 | **0.00462** |
|  |  | s(Light): Baseline | 1.931 | 1.995 | 14.44 | **1.06e-05** |
|  |  | s(Light): Hawaii | 1.964 | 1.999 | 22 | **3.30e-07** |
|  |  | s(Light): Texas | 1.983 | 2 | 30.36 | **< 2e-16** |
|  |  | s(Light): California | 1.948 | 1.997 | 29.69 | **< 2e-16** |
|  |  | s(Light): New Mexico | 1.874 | 1.984 | 12.59 | **3.23e-05** |
|  | 25°C | Site | 4 | - | 3.399 | **0.0151** |
|  |  | s(Light) | 1.964 | 1.999 | 23.39 | **< 2e-16** |
|  | 30°C | Site | 4 | - | 4.203 | **0.00539** |
|  |  | s(Light): Baseline | 1.591 | 1.833 | 2.881 | 0.131 |
|  |  | s(Light): Hawaii | 1.297 | 1.506 | 2.15 | 0.208 |
|  |  | s(Light): Texas | 1 | 1 | 4.612 | **0.0368** |
|  |  | s(Light): California | 1.887 | 1.987 | 4.437 | **0.0204** |
|  |  | s(Light): New Mexico | 1.556 | 1.803 | 1.237 | 0.205 |
| summer | 5°C | Site | 3 | - | 3.312 | **0.0297** |
|  |  | s(Light): Baseline | 1.714 | 1.918 | 11.881 | **4.21e-04** |
|  |  | s(Light): Hawaii | 1 | 1 | 9.646 | **3.53e-03** |
|  |  | s(Light): Texas | 1.095 | 1.182 | 7.393 | **0.00956** |
|  |  | s(Light): California | 1 | 1 | 0.014 | 0.90668 |
|  | 10°C | Site | 3 | - | 9.691 | **7.19e-05** |
|  |  | s(Light): Baseline | 1 | 1 | 36.499 | **6.58e-07** |
|  |  | s(Light): Hawaii | 1.79 | 1.956 | 5.938 | **3.90e-03** |
|  |  | s(Light): Texas | 1.779 | 1.951 | 1.677 | 0.18882 |
|  |  | s(Light): California | 1.916 | 1.993 | 6.151 | **0.00409** |
|  | 15°C | Site | 3 | - | 11.78 | **1.33e-05** |
|  |  | s(Light): Baseline | 1.902 | 1.99 | 36.039 | **< 2e-16** |
|  |  | s(Light): Hawaii | 1.001 | 1.002 | 18.671 | **1.07e-04** |
|  |  | s(Light): Texas | 1.499 | 1.749 | 0.403 | 0.63805 |
|  |  | s(Light): California | 1.537 | 1.785 | 6.851 | **0.00258** |
|  | 20°C | Site | 3 | - | 1.806 | 0.163 |
|  |  | s(Light): Baseline | 1.978 | 2 | 28.44 | **< 2e-16** |
|  |  | s(Light): Hawaii | 1.969 | 1.999 | 17.45 | **4.70e-06** |
|  |  | s(Light): Texas | 1.911 | 1.992 | 11.7 | **0.00021** |
|  |  | s(Light): California | 1.944 | 1.997 | 9.08 | **0.00057** |
|  | 25°C | Site | 3 | - | 6.294 | **0.00142** |
|  |  | s(Light): Baseline | 1.517 | 1.767 | 0.83 | 0.316 |
|  |  | s(Light): Hawaii | 1 | 1 | 25.42 | **1.18e-05** |
|  |  | s(Light): Texas | 1.928 | 1.995 | 35.28 | **< 2e-16** |
|  |  | s(Light): California | 1.51 | 1.76 | 21.11 | **1.12e-05** |
|  | 30°C | s(Light) | 1 | 1 | 10.77 | **0.002** |

**Site indicates the parametric term in GAM, s(Light) is the smooth term for each site. df/edf column indicates either df (degrees of freedom) for parametric terms or edf (effective degrees of freedom) for smoother terms; Ref.df = reference degree of freedom, where dashes indicate NA for parametric terms. Significant effects (p<0.05) are in bold. No summer data is shown for New Mexico culture due to health issues during the acclimation process.*

**Table S7.** Model selection for light performance curve comparison between seasons with candidate GAM models assessed at each temperature treatment, corresponding to 5, 10, 15, 20, 25, and 30°C.

| Site | Temperature | Model | df | AIC |
| --- | --- | --- | --- | --- |
| Baseline | 5°C | **~Season + s(Light, by= Season)** | **6.9** | **-166.9** |
|  |  | ~Season + s(Light) | 4 | -151.9 |
|  |  | ~s(Light) | 3 | -131.6 |
|  | 10°C | **~Season + s(Light, by= Season)** | **5.8** | **-158.7** |
|  |  | ~Season + s(Light) | 4 | -133.2 |
|  |  | ~s(Light) | 3 | -129.7 |
|  | 15°C | **~Season + s(Light, by= Season)** | **7** | **-163.8** |
|  |  | ~Season + s(Light) | 5 | -154 |
|  |  | ~s(Light) | 4 | -155.9 |
|  | 20°C | **~Season + s(Light, by= Season)** | **7** | **-158.7** |
|  |  | ~Season + s(Light) | 5 | -135.8 |
|  |  | ~s(Light) | 4 | -132.9 |
|  | 25°C | ~Season + s(Light, by= Season) | 6.8 | -144.4 |
|  |  | **~Season + s(Light)** | **5** | **-147.2** |
|  |  | ~s(Light) | 4 | -144.3 |
|  | 30°C | **~Season + s(Light, by= Season)** | **5.9** | **-181** |
|  |  | ~Season + s(Light) | 4 | -152.7 |
|  |  | ~s(Light) | 3 | -147.7 |
| Hawaii | 5°C | **~Season + s(Light, by= Season)** | **6** | **-165.2** |
|  |  | ~Season + s(Light) | 4.8 | -153.3 |
|  |  | ~s(Light) | 3.6 | -143.7 |
|  | 10°C | **~Season + s(Light, by= Season)** | **6.5** | **-172.8** |
|  |  | ~Season + s(Light) | 5 | -157.4 |
|  |  | ~s(Light) | 3.6 | -131.8 |
|  | 15°C | **~Season + s(Light, by= Season)** | **6.8** | **-168.6** |
|  |  | ~Season + s(Light) | 5 | -160.8 |
|  |  | ~s(Light) | 4 | -156.9 |
|  | 20°C | **~Season + s(Light, by= Season)** | **7** | **-143.3** |
|  |  | ~Season + s(Light) | 5 | -140.1 |
|  |  | ~s(Light) | 4 | -130.4 |
|  | 25°C | **~Season + s(Light, by= Season)** | **6** | **-144.8** |
|  |  | ~Season + s(Light) | 4.1 | -116.4 |
|  |  | ~s(Light) | 3 | -116.4 |
|  | 30°C | **~Season + s(Light, by= Season)** | **5.8** | **-189.5** |
|  |  | ~Season + s(Light) | 4 | -163.7 |
|  |  | ~s(Light) | 3 | -157.6 |
| Texas | 5°C | **~Season + s(Light, by= Season)** | **6.2** | **-173** |
|  |  | ~Season + s(Light) | 4.8 | -156 |
|  |  | ~s(Light) | 3.8 | -157.9 |
|  | 10°C | ~Season + s(Light, by= Season) | 5.9 | -152.2 |
|  |  | **~Season + s(Light)** | **4.9** | **-152.5** |
|  |  | ~s(Light) | 3.9 | -149.7 |
|  | 15°C | **~Season + s(Light, by= Season)** | **6.8** | **-148.1** |
|  |  | ~Season + s(Light) | 5 | -140.5 |
|  |  | ~s(Light) | 4 | -141.9 |
|  | 20°C | **~Season + s(Light, by= Season)** | **7** | **-124.9** |
|  |  | ~Season + s(Light) | 5 | -117.6 |
|  |  | ~s(Light) | 4 | -119.3 |
|  | 25°C | **~Season + s(Light, by= Season)** | **7** | **-138.8** |
|  |  | ~Season + s(Light) | 5 | -115.6 |
|  |  | ~s(Light) | 4 | -117.7 |
|  | 30°C | ~Season + s(Light, by= Season) | 5 | -144.3 |
|  |  | ~Season + s(Light) | 4 | -146.1 |
|  |  | **~s(Light)** | **3** | **-146.8** |
| California | 5°C | **~Season + s(Light, by= Season)** | **6** | **-165.7** |
|  |  | ~Season + s(Light) | 4.8 | -157.6 |
|  |  | ~s(Light) | 3.6 | -145.9 |
|  | 10°C | **~Season + s(Light, by= Season)** | **6** | **-181.8** |
|  |  | ~Season + s(Light) | 5 | -169.8 |
|  |  | ~s(Light) | 3.6 | -118.5 |
|  | 15°C | ~Season + s(Light, by= Season) | 6.1 | -140.3 |
|  |  | **~Season + s(Light)** | **4.9** | **-142.1** |
|  |  | ~s(Light) | 3.8 | -136.8 |
|  | 20°C | **~Season + s(Light, by= Season)** | **7** | **-132.1** |
|  |  | ~Season + s(Light) | 5 | -129.5 |
|  |  | ~s(Light) | 4 | -115.7 |
|  | 25°C | **~Season + s(Light, by= Season)** | **6.7** | **-136.4** |
|  |  | ~Season + s(Light) | 4.8 | -116.5 |
|  |  | ~s(Light) | 3.8 | -118.3 |
|  | 30°C | ~Season + s(Light, by= Season) | 5.5 | -111.5 |
|  |  | ~Season + s(Light) | 4 | -111.1 |
|  |  | **~s(Light)** | **3** | **-112.5** |

**Model 3: Season + s(*Light*, by= Season) GAM has parametric terms (Season) and separate smoothers for each season. Model 2: Season + s(*Light*) GAM has a global smoother allowing for offset intercepts according to season. Model 1: s(*Light*) GAM fits a global smoother to all data. Bold represents the selected models. No data is shown for New Mexico culture due to health issues during the summer acclimation process.*

**Table S8.** Generalized additive models (GAM) testing season and factor-smooth interaction effects on growth rate measured at each site for light performance curve. Separate smoothers were fit for each season, and ANOVA tables were generated by anova.gam().

| Site | Temperature | Effect | df/edf | Ref.df | F | p-value |
| --- | --- | --- | --- | --- | --- | --- |
| Baseline | 5°C | Season | 1 | - | 67.05 | **1.47e-07** |
|  |  | s(Light): winter | 1.899 | 1.990 | 5.539 | **0.01619** |
|  |  | s(Light): summer | 1.648 | 1.876 | 9.894 | **0.00353** |
|  | 10°C | Season | 1 | - | 16.93 | **0.000564** |
|  |  | s(Light): winter | 1.506 | 1.756 | 5.629 | **0.031** |
|  |  | s(Light): summer | 1.000 | 1.000 | 44.679 | **9.79e-07** |
|  | 15°C | Season | 1 | - | 0.02 | 0.89 |
|  |  | s(Light): winter | 1.941 | 1.997 | 29.76 | **8.91e-07** |
|  |  | s(Light): summer | 1.955 | 1.998 | 81.53 | **< 2e-16** |
|  | 20°C | Season | 1 | - | 12.31 | **0.0025** |
|  |  | s(Light): winter | 1.950 | 1.997 | 20.21 | **1.99e-05** |
|  |  | s(Light): summer | 1.994 | 2.000 | 106.40 | **< 2e-16** |
|  | 25°C | Season | 1 | - | 4.535 | **0.0457** |
|  |  | s(Light) | 1.850 | 1.978 | 6.818 | **0.00403** |
|  | 30°C | Season | 1 | - | 24.78 | **7.93e-05** |
|  |  | s(Light): winter | 1.646 | 1.875 | 3.313 | 0.107 |
|  |  | s(Light): summer | 1.000 | 1.000 | 69.091 | **< 2e-16** |
| Hawaii | 5°C | Season | 1 | - | 21.52 | **0.000176** |
|  |  | s(Light): winter | 1.876 | 1.985 | 5.969 | **0.0138** |
|  |  | s(Light): summer | 1.000 | 1.000 | 7.019 | **0.0158** |
|  | 10°C | Season | 1 | - | 87.83 | **1.61e-08** |
|  |  | s(Light): winter | 1.270 | 1.467 | 2.353 | 0.19 |
|  |  | s(Light): summer | 1.907 | 1.991 | 14.945 | **9.52e-05** |
|  | 15°C | Season | 1 | - | 8.168 | **0.0103** |
|  |  | s(Light): winter | 1.961 | 1.998 | 65.33 | **< 2e-16** |
|  |  | s(Light): summer | 1.594 | 1.835 | 25.72 | **5.85e-06** |
|  | 20°C | Season | 1 | - | 15.32 | **0.00101** |
|  |  | s(Light): winter | 1.951 | 1.998 | 15.57 | **9.89e-05** |
|  |  | s(Light): summer | 1.984 | 2.000 | 34.32 | **< 2e-16** |
|  | 25°C | Season | 1 | - | 6.691 | **0.018** |
|  |  | s(Light): winter | 1.851 | 1.978 | 9.499 | **0.00101** |
|  |  | s(Light): summer | 1.000 | 1.000 | 34.432 | **9.58e-06** |
|  | 30°C | Season | 1 | - | 26.25 | **5.65e-05** |
|  |  | s(Light): winter | 1.572 | 1.817 | 3.381 | 0.109 |
|  |  | s(Light): summer | 1.000 | 1.000 | 56.592 | **< 2e-16** |
| Texas | 5°C | Season | 1 | - | 0.21 | 0.652 |
|  |  | s(Light): winter | 1.941 | 1.997 | 9.896 | **0.00134** |
|  |  | s(Light): summer | 1.111 | 1.209 | 7.409 | **0.01344** |
|  | 10°C | Season | 1 | - | 4.465 | **0.0472** |
|  |  | s(Light) | 1.756 | 1.941 | 2.161 | 0.18 |
|  | 15°C | Season | 1 | - | 0.69 | 0.417 |
|  |  | s(Light): winter | 1.944 | 1.997 | 15.783 | **8.96e-05** |
|  |  | s(Light): summer | 1.563 | 1.809 | 0.553 | 0.558 |
|  | 20°C | Season | 1 | - | 0.276 | 0.606 |
|  |  | s(Light): winter | 1.948 | 1.997 | 9.711 | **0.00127** |
|  |  | s(Light): summer | 1.900 | 1.990 | 10.511 | **0.00141** |
|  | 25°C | Season | 1 | - | 0.001 | 0.979 |
|  |  | s(Light): winter | 1.923 | 1.994 | 6.274 | **0.00778** |
|  |  | s(Light): summer | 1.934 | 1.996 | 38.279 | **< 2e-16** |
|  | 30°C | s(Light) | 1 | 1 | 1.292 | 0.268 |
| California | 5°C | Season | 1 | - | 22.88 | **0.000128** |
|  |  | s(Light): winter | 1.920 | 1.994 | 6.762 | **0.00708** |
|  |  | s(Light): summer | 1.000 | 1.000 | 0.010 | 0.92109 |
|  | 10°C | Season | 1 | - | 282.2 | **7.22e-13** |
|  |  | s(Light): winter | 1.000 | 1.000 | 6.039 | **0.0238** |
|  |  | s(Light): summer | 1.974 | 1.999 | 21.351 | **1.1e-05** |
|  | 15°C | Season | 1 | - | 7.156 | **0.0144** |
|  |  | s(Light) | 1.687 | 1.902 | 18.16 | **2.81e-05** |
|  | 20°C | Season | 1 | - | 22.15 | **0.000172** |
|  |  | s(Light): winter | 1.885 | 1.987 | 12.67 | **0.000275** |
|  |  | s(Light): summer | 1.954 | 1.998 | 11.14 | **0.000650** |
|  | 25°C | Season | 1 | - | 0.34 | 0.567 |
|  |  | s(Light): winter | 1.697 | 1.908 | 2.924 | 0.0541 |
|  |  | s(Light): summer | 1.507 | 1.757 | 21.020 | **7.5e-05** |
|  | 30°C | s(Light) | 1 | 1 | 0.892 | 0.356 |

**Season indicates the parametric term in GAM, s(Light) is the smooth term for each season. df/edf column indicates either df (degrees of freedom) for parametric terms or edf (effective degrees of freedom) for smoother terms; Ref.df = reference degree of freedom, where dashes indicate NA for parametric terms. Significant effects (p<0.05) are in bold. No data is shown for New Mexico culture due to health issues during the summer acclimation process.*
